# Geographical pattern of genetic diversity in *Capsella bursa‐pastoris* (Brassicaceae)—A global perspective

**DOI:** 10.1002/ece3.7010

**Published:** 2020-11-13

**Authors:** Christina Wesse, Erik Welk, Herbert Hurka, Barbara Neuffer

**Affiliations:** ^1^ Department of Botany University of Osnabrueck Osnabrück Germany; ^2^ Institute for Biology Martin‐Luther‐University Halle‐Wittenberg Halle (Saale) Germany

**Keywords:** adaptation, *Capsella bursa‐pastoris*, colonization, macroclimate, multilocus genotypes, species distribution model

## Abstract

We analyzed the global genetic variation pattern of *Capsella bursa‐pastoris* (Brassicaceae) as expressed in allozymic (within‐locus) diversity and isozymic (between‐locus) diversity. Results are based on a global sampling of more than 20,000 *C. bursa‐pastoris* individuals randomly taken from 1,469 natural provenances in the native and introduced range, covering a broad spectrum of the species’ geographic distribution. We evaluated data for population genetic parameters and *F*‐statistics, and Mantel tests and AMOVA were performed. Geographical distribution patterns of alleles and multilocus genotypes are shown in maps and tables. Genetic diversity of introduced populations is only moderately reduced in comparison with native populations. Global population structure was analyzed with *structure*, and the obtained cluster affiliation was tested independently with classification approaches and macroclimatic data using species distribution modeling. Analyses revealed two main clusters: one distributed predominantly in warm arid to semiarid climate regions and the other predominantly in more temperate humid to semihumid climate regions. We observed admixture between the two lineages predominantly in regions with intermediate humidity in both the native and non‐native ranges. The genetically derived clusters are strongly supported in macroclimatic data space. The worldwide distribution patterns of genetic variation in the range of *C. bursa‐pastoris* can be explained by intensive intra‐ and intercontinental migration, but environmental filtering due to climate preadaption seems also involved. Multiple independent introductions of genotypes from different source regions are obvious. “Endemic” genotypes might be the outcome of admixture or of de novo mutation. We conclude that today's successfully established *Capsella* genotypes were preadapted and found matching niche conditions in the colonized range parts.

## Graphical abstract

In the current literature, the colonization history of Shepherd’s Purse (*Capsella bursa‐pastoris*) has been traced by molecular markers for certain regions. However, a worldwide consideration of the entire range of the species is still missing. Here, we provide the worldwide distribution patterns of genetic variation that can be explained by environmental filtering due to climate preadaptation.

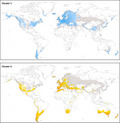

## 1 INTRODUCTION

Colonization and range expansion are basic features of the evolutionary history of all species and may occur over geological timescales to more recent man‐caused dispersal, from intercontinental migration to regional and local range extensions. The use of a diverse array of neutral molecular markers, for example, isozymes, RAPDs, AFLPs, microsatellites, and finally DNA sequences and next‐generation sequencing, has greatly enhanced the ability to reconstruct the evolutionary history of colonization processes and to assess the magnitude of genetic bottlenecks and founder events (e.g., Barrett, 2015; Cristescu, 2015). There is now evidence from neutral loci that many populations of introduced species have less genetic variation than populations in the native range (Barrett, 2015), although the genetic diversity of introduced, non‐native populations seems to be only moderately reduced in comparison with native populations (Bossdorf et al., 2005; Dlugosch & Parker, 2008). However, inferences regarding differentiation between genotypes from the native to the introduced range are prone to sampling errors and i.e., are often confounded by nonrandom geographic sampling, thus missing among‐population variation within each range when diversity is geographically structured (Colautti & Lau, 2015). In the present study, we analyze genetic data that offer insights into patterns of global population differentiation: the sources, routes, and global spread of one of the most frequent and widespread flowering plants on earth *Capsella bursa‐pastoris* (L.) Medik., Shepherd's Purse (Brassicaceae).

*Capsella bursa‐pastoris* originated in Eurasia probably in pre‐(Weichselian) glacial times (Douglas et al., 2015; Hurka et al., 2012; Hurka & Neuffer, 1997). *C. bursa‐pastoris* is tetraploid (2*n* = 32), and its putative parental lineages are ancestral to present‐day *Capsella orientalis* (distributed predominantly in temperate continental West Asia) and *Capsella grandiflora* (distributed mainly in western Greece) (Douglas et al., 2015; Hurka et al., 2012). In post‐Columbian time, European colonists started introducing *C. bursa‐pastoris* to the New World, Australasia, and southern Africa. In a number of studies, molecular markers have been used to trace regional colonization histories (e.g., RAPDs in Neuffer, 1996; isozymes in Neuffer & Hurka, 1999; isozymes and RAPDs in Neuffer et al., 1999; isozymes in Neuffer et al., 2011; low‐copy nuclear gene fragments in Han et al., 2015; genotyping‐by‐sequencing/SNPs in Cornille et al., 2016, and Kryvokhyzha et al., 2019). The analyses by Cornille et al. (2016) suggest a probably multistage colonization history starting from the Eastern Mediterranean to Europe in the middle to late Pleistocene. The latest expansion toward East Asia seems to have been directly human related and is dated as rather rapid and recent (ca. 1,000 years). The objective of the present study was to display and analyze the global genetic variation pattern of *C. bursa‐pastoris* as expressed in isozyme variability. Is genetic variation spatially structured in the native range and mirrored in the introduced range and, if so, how can this be explained? Specifically, we ask whether the geographic pattern of genetic variation is mainly driven by colonization via increasing human transport activities, which should result in a random pattern. Or, whether a certain degree of environmental filtering is detectable, this should be reflected in/by ecologically structured genetic variation.

## 2 MATERIAL AND METHODS

The meta‐data set comprises >20,000 individuals from an array of provenances covering large distribution areas in the native and the introduced ranges. We evaluated the data in rising complexity, from allozyme frequencies via single‐locus up to multilocus genotypes (*F*‐statistics, Mantel's test, AMOVA). Informative value increases along this sequence. Finally, to assess the structure of allele frequency variation in the allozyme data set, we used clustering approaches to assign multilocus genotypes into clusters and species distribution modeling to predict potential distribution of the clusters in relation to macroclimatic data.

### 2.1 Plant Material

Individual seed samples from *C. bursa‐pastoris* plants were collected from 1,469 natural provenances from all over the world. Samples were geolocated and assigned to geographical regions (Table S1a): *Iberian Peninsula* (IBE): Portugal and Spain; *British Isles* (BRT): Great Britain and Ireland; *Middle and Western Europe* (M + WE): Andorra, Austria, Czech Republic, France, Germany, Hungary, Liechtenstein, Netherlands, Poland, Slovakia, and Switzerland; *Mediterranean areas* (MED): Egypt, Greece, Israel, Italy, Morocco, Turkey, and former Yugoslavia; *Scandinavia* (SCN): Denmark, Finland, Iceland, Norway, and Sweden; *Eastern Europe* (EEU): Bulgaria, Estonia, and European Russia; *Asia* (ASIA): Afghanistan, Armenia, Asian Russia, China, Iran, Japan, Kazakhstan, Kyrgyzstan, Mongolia, Nepal, and Sri Lanka; *California* (CAL); *North America* (NAM): Canada and USA (except California); and *Middle and South America* (M + SA): Argentina, Bolivia, Chile, Costa Rica, Ecuador, Falkland Islands, Mexico, and Venezuela; and *Australasia* (AUS): Australia and New Zealand; and *Africa* (AFR): Republic of South Africa.

Seeds were stored in plastic bags at −20°C until sowing for analyses. Determination of species was performed with cultivated flowering individuals, chromosome counting, flow cytometry, and isozyme analysis. Isozymes facilitate the distinction between tetraploid and diploid individuals, which occasionally might otherwise be difficult. 27,323 individuals from the whole genus were used in this study, with *n* = 21,812 identified as *C. bursa‐pastoris*, *n* = 263 as *Capsella thracica* Velen., *n* = 109 as *C. orientalis*, *n* = 3,141 as *Capsella rubella* Reuter, and *n* = 1,998 as *C. grandiflora* (see Table S2). Herbarium material of many accessions is deposited in the Herbarium of the University of Osnabrueck OSBU. Plants were grown in the greenhouse of the Department of Botany or the Botanical Garden of the University Osnabrueck, and rosette leaves of single plants were harvested and stored at −80°C.

### 2.2 Isozyme analyses

Electrophoresis was performed in a continuous system on vertical polyacrylamide gel slabs (PAGE). Following isozyme systems were assayed: aspartate aminotransferase (AAT; EC2.6.1.1), glutamate dehydrogenase (GDH; EC1.4.1.4), and leucine aminopeptidase (LAP; 3.4.11.1). For buffer systems and other experimental details, see Hurka et al., (1989) for AAT, Hurka and Düring (1994) for GDH, and Neuffer and Hurka (1999) for LAP. Isozyme data were either previously published or are presented here for the first time. The genetics of these enzyme systems in *Capsella* have been deciphered in the above‐cited literature, and the previous nomenclature of the enzyme loci and their isozymes is adopted in the present study with few modifications: Re‐evaluation of the isozyme patterns motivated us to include the former alleles *Lap3‐1* into *Lap3‐2*, *Lap3‐3* into *Lap3‐4*, and *Lap3‐7* into *Lap3‐2*.

### 2.3 Data evaluation

*Capsella bursa‐pastoris* is tetraploid and thus comprises two complete genomes, A and B. Each of the single loci is doubled in *C. bursa‐pastoris* and constitutes a locus pair with four alleles, two from genome A and two from genome B. Inheritance is disomic (Shull, 1929 for morphological characters; Hurka & Düring, 1994; Hurka et al., 1989; Hurka & Neuffer, 1997 for allozymes). Since it is not possible to assign each of the four alleles of a locus pair unambiguously to one of the two loci of a pair, we recorded the presence or absence of the different alleles at each locus pair.

Data were evaluated with the program GenAlEx 6 (Peakall & Smouse, 2006, 2012).

We analyzed allele frequencies and genotype frequencies at single and multiple loci in total, and within and between regions. Measurements of genetic variation, F‐statistics, and Mantel's test were performed. Significance for the Mantel test was based on 999 permutations. For multilocus samples without missing data (*n* = 8,076), we quantified population genetic diversity of *C. bursa‐pastoris* using an analysis of molecular variance (AMOVA) (Excoffier et al., 1992 implemented in GenAlEx 6) and calculated a measure of genetic variation (SSWP/*n* − 1) by calculating the population‐wise AMOVA sums of squares divided by *n* − 1 (Fischer & Matthies, 1998). The SSWP values were sample size‐corrected.

### 2.4 Global genotype classification

We used a Bayesian clustering method to find an optimal and robust partition of the sampled populations based on isozyme data. First, we quantified global population structure in the data set with the software *structure* 2.3.4 (Pritchard et al., 2000). For each analysis, we implemented a model of correlated allele frequencies (Falush et al., 2003) and admixture, and applied the default setting for all other parameters. Ten independent runs for values of *K* (number of genetic clusters) between 1 and 10 were performed using an MCMC length of 10^6^ generations following a burn‐in of 10^5^ generations. For each *K* value, we used *clumpp* v. 1.1.2 software (Jakobsson & Rosenberg, 2007) to examine consistency across replicate cluster analyses by estimating the highest value of pairwise similarity (*H*′ value) and averaged assignment probabilities for each individual. We applied the *Greedy* algorithm for *K* = 1–10, using 1,000 random input orders. The most probable *K* value was chosen by examining the log probability of the data [ln Pr(*X*|*K*)] and plots of ∆*K* (Evanno et al., 2005) obtained by using the software *structure harvester* (Earl & von Holdt, 2012). To further test the robustness of the *structure* partitioning, an alternative hierarchical clustering approach was adopted with several distance measures for binary data in the R package ade4 (Dray et al., 2015) and procedures for the determination of optimal cluster solutions with the R package *starmie* (Tonkin‐Hill & Lee, 2017). To assess alternative clustering solutions, the partitionings for *K* = 3 and *K* = 4 were evaluated for possible ecogeographical interpretations.

### 2.5 Classification tree analysis

To test whether independent macroclimatic data would support the cluster structure derived from isozyme pattern, classification tree analyses were performed. Specifically, we tested whether and which bioclimatic variables supported the distinction of the found population groups for *K* = 2–4. Prior to any modeling steps, collinearity diagnostics were performed to avoid detrimental effects of multicollinearity and variance inflation. Specifically, we calculated a pairwise correlation matrix to identify collinearities that exist among the 19 bioclimatic predictors. In highly correlated predictor pairs (Pearson *r* > 0.75) one variable was excluded, respectively. This procedure retained seven variables (Bio2, Bio3, Bio6, Bio8, Bio13, Bio15, and Bio18). Since the obtained clusters are similar in population samples, no resampling was necessary.

Several alternative classification approaches were adopted to enable consideration of methodological differences. First, a simple classification tree analysis was performed using the *rpart* package in R (Therneau & Atkinson, 2019). Second, *party*—a recursive partitioning approach with conditional inference trees—was adopted (Strobl et al., 2008). Third, random forest ensembles of recursive classification trees were adopted with the *randomForest* package in R (Liaw & Wiener, 2002). As settings for the latter we used 5,000 iterations and the *tuneRF* function to identify the “optimal” number of input variables randomly chosen at each node. Classification success was measured as prediction accuracy based on confusion matrices.

### 2.6 Distribution modeling of genotype clusters (SDM)

Because *C. bursa‐pastoris* is a cosmopolitan species, known to be present at various locations around the world, a species distribution modeling (SDM) approach was used to predict and analyze the potential distribution of the two clusters derived from the population genetic clustering approach (*structure*). To this end, the cluster affiliation probabilities from the population genetic structure analysis were binarized at the threshold of 0.5 probability for the respective cluster. We used the software *MaxEnt* v. 3.3.4, which provides a machine learning SDM algorithm (Phillips et al., 2006). Unlike presence–absence approaches, the MaxEnt algorithm is based primarily on presence data as the basis of its predictions and is therefore especially suitable for the given data since absence data for genotypes are not available. *MaxEnt* has repeatedly been proven to be an effective method for predicting potential species distributions in scarce data situations (e.g., Merow et al., 2013). Elith et al. (2006) found that *MaxEnt* was one of the best of 16 different methods for modeling the distributions of 226 species in 6 different regions.

Climate data at the population sampling localities were extracted as part of the MaxEnt distribution modeling with removal of duplicate points per raster grid cell. We made use of the “WorldClim version 2” database (Fick & Hijmans, 2017) that is available for download from http://worldclim.org/. As environmental variables, we used Bioclim temperature metrics (Bio01‐11) and precipitation metrics (Bio12‐19) at a spatial resolution of 2.5 arc minutes (approximately 4.5 km^2^ at the equator). The iteratively self‐optimizing *MaxEnt* algorithm inherently identifies variables that contribute most to increasing predictive success. Thus, an a priori variable exclusion via cross‐correlation or PCA approaches is not mandatory, and one of two potentially correlated variables will be down weighted in the prediction process. The R package *ENMeval* (Muscarella et al., 2014) was used to maximize predictive ability and avoid overfitting problems that might result from the spatial clustering of sampling localities (see Radosavljevic & Anderson, 2014).

From the *ENMeval* model comparison approach, the best resulting model configuration in terms of complexity (AICc) and accuracy (AUC) was selected for each cluster, respectively. Accordingly, we performed multiple runs, with random fivefold cross‐validation between test and training data. Linear, quadratic, and polynomial functions (L, Q, and P) were used as single and combined response options, and the number of background samples was set to 50,000 to enable the worldwide climatic background to be sampled. A regularization value of 1.1 was obtained as the best option to avoid overfitting. Finally, the resulting model solutions were compared in terms of complexity (AICc) and accuracy (AUC), and the best resulting models were selected for each cluster, respectively.

## 3 RESULTS

### 3.1 Allele biogeography

**Figure 1:**
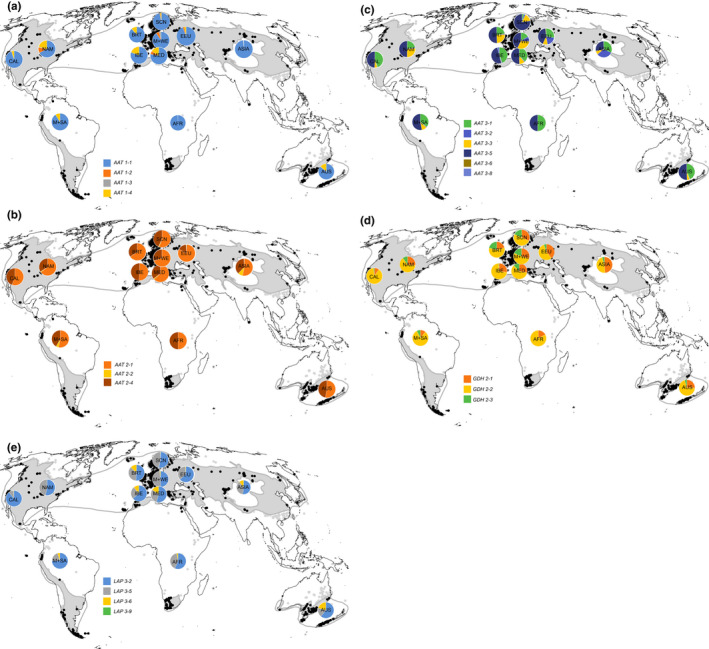
(a) Allele frequencies of *Capsella bursa‐pastoris*in different regions (Locus*Aat1*). (b) Allele frequencies of*C. bursa‐pastoris*in different regions (Locus*Aat2*). (c) Allele frequencies of*C. bursa‐pastoris*in different regions (Locus*Aat3*). (d) Allele frequencies of *C. bursa‐pastoris*in different regions (Locus*Gdh2*). (e) Allele frequencies of*Capsella bursa‐pastoris*in different regions (Locus*Lap3*).AFR, Africa; AUS, Australasia; BRT, British Isles; CAL, California; EEU, Eastern Europe; IBE, Iberian Peninsula; M + SA, Middle and South America; M + WE, Middle and Western Europe; MED, circum‐Mediterranean region; NAM, North America except California. Black dots: sample locations, grey areas: distribution of*C. bursa‐pastoris* based on data compiled by EW (CDH, 2018)

Allele frequencies in different regions are pictured in Figure 1a–e. Absolute numbers of alleles and their frequencies in percent between and within regions are presented in Table S3.

### 3.2 Genotype biogeography

#### 3.2.1 Loci and loci associations

**Figure 2:**
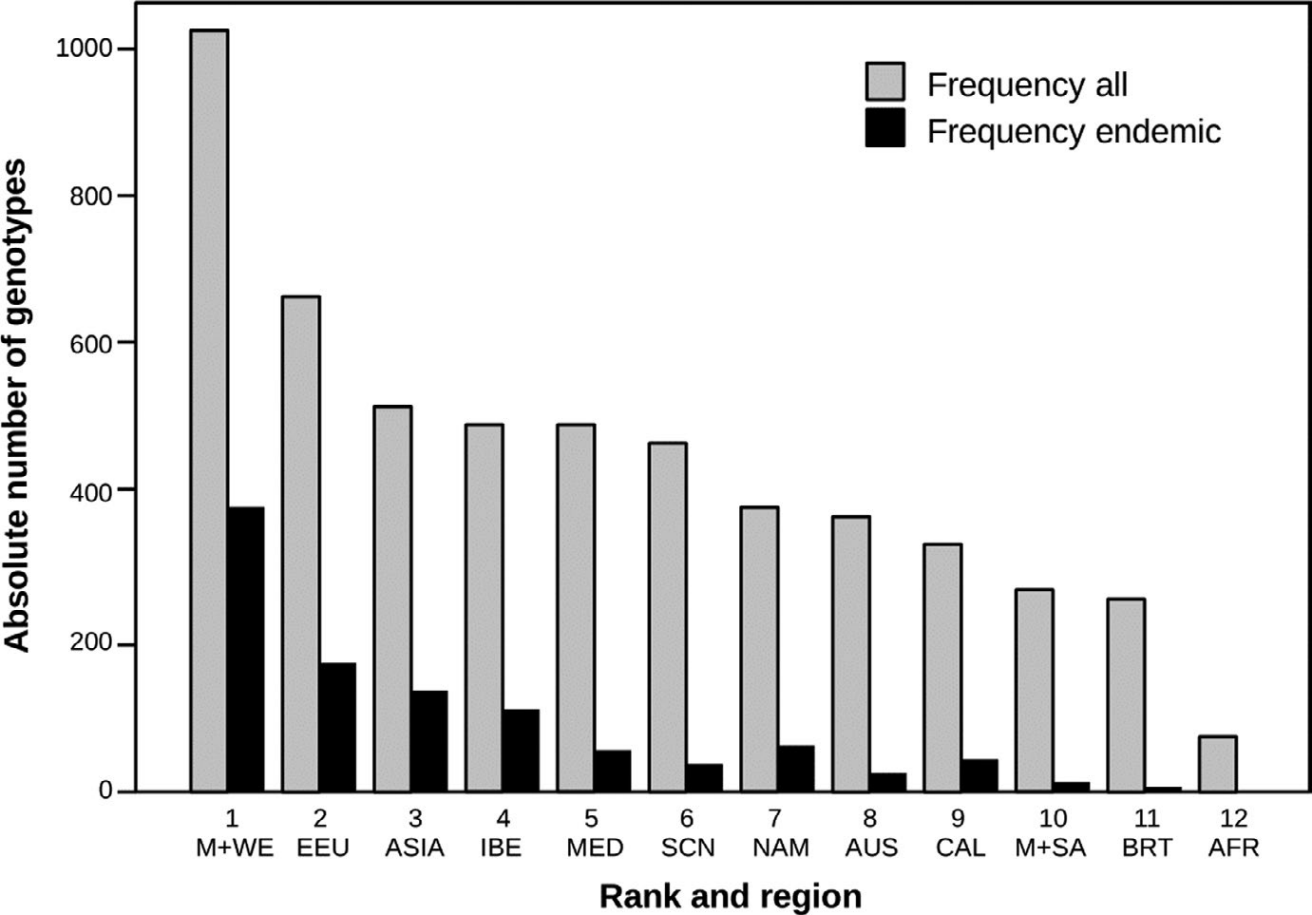
Rank statistics of genotype diversity of *Capsella bursa‐pastoris* within different regions. Order 1: highest diversity, order 12: lowest diversity. AFR, Africa; AUS, Australasia; BRT, British Isles; CAL, California; EEU, Eastern Europe; IBE, Iberian Peninsula; M + SA, Middle and South America; M + WE, Middle and Western Europe; MED, circum‐Mediterranean region; NAM, North America except California

We analyzed genotype frequencies at single loci and at different associations in total and in the geographical regions (Table S4). Altogether, 1,851 different genotype combinations have been detected. Frequencies of given genotypes differ significantly between regions and are mostly rather low. Only 66 out of the 1,851 recorded genotypes show frequencies of 10% and higher (hereafter referred to as “frequent genotypes”). It is obvious that frequent genotypes are preferentially shared by certain regions, for example, IBE and MED with CAL and AUS, M + WE with SCN and EEU, and M + WE with IBE and MED (Table S4). If we plot regional presence of genotypes irrespective of their frequencies, we observe differences in regional genotype diversity: Transforming the nominal scale into order statistics reveals an interesting rank order of the geographical regions (Figure 2). M + WE is the most diverse region (rank order 1) harvesting ca. 55% of the total sum of genotypes. It is followed by EEU (rank order 2) with 35% of the total, and ASIA, IBE, MED, and SCN with 28%–25%, approximately half that of M + WE and more or less equal between these regions. Genotype diversity within the introduced range is significantly lower than in the native ranges, displaying only 20% and less of the total genotypes. AFR, with only 67 different genotypes, occupies the last position (rank order 12). A remarkable exception is BRT from the native range with the penultimate position (rank order 11) (Figure 2). The number of genotypes found is slightly correlated to the number of samples taken (Pearson's *r* = 0.62, *p* = .03).

#### 3.2.2 The complete multilocus association

Out of all loci associations analyzed, we focus here on complete loci associations with the locus sequence *Aat1*, *Aat2*, *Aat3*, *Gdh1*, *Gdh2*, and *Lap3*. A total of 8,076 individuals recorded in the native and non‐native ranges provided this complete multilocus combination (Table S5a,b). We detected 383 different genotypes at this multilocus, and only 18 of them had frequencies >1% out of which only one was frequent (*f* = 18%, Table S5a,b). 5,658 individuals shared these genotypes with frequencies >1%, whereas 2,418 individuals displayed rare genotypes. All of the 18 common complete multilocus genotypes were recorded from the native and the introduced ranges but with different frequencies between and within the different geographical regions (Table S5a,b). Of particular interest is the so‐called Mediterranean multilocus genotype (MMG) with the composition *Aat1‐1111*, *Aat2‐1144*, *Aat3‐1155*, *Gdh1‐1111*, *Gdh2‐2222*, and *Lap3‐2222* (Neuffer & Hoffrogge, 1999; Neuffer & Hurka, 1999). In the native range, the MMG occurs predominantly in the Iberian Peninsula, and in the introduced ranges with high frequencies in California where it is the most common genotype (Table S5a,b). It is also rather frequent in Middle and South America and in Australasia (Table S5a,b) and contributes remarkably to the set of multilocus genotypes in these regions (Table S5a,b).

#### 3.2.3 “Endemic” genotypes

In nearly all geographical regions, some of the recorded genotypes were “endemic” which means they were not recorded in any other region (Figure 2, Table S6). No “endemic” genotypes were detected in AFR. Worth mentioning is also BRT with only four “endemics” out of the total 250 genotypes (i.e., <2%). “Endemism” is low also in M + SA, SCN, and AUS (ca. 4%–8%). In the other regions, the proportion of “endemics” ranges between 12% and 37%. While the number of genotypes found was positively related to the number of samples taken (Pearson's *r* = 0.62, *p* = .03), endemism calculated as the ratio of endemic to overall genotypes was unrelated to sampling intensity (Pearson's *r* = 0.52, *p* = .085).

### 3.3 Population structure analysis

**Figure 3:**
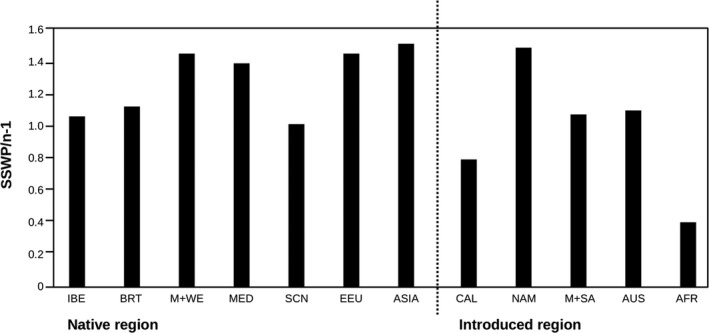
SSWP/n ‐ 1 diversity values of *Capsella bursa‐pastoris* populations within the native (pre‐Columbian) and introduced (post‐Columbian) regions. AMOVA tested for differences among groups. IBE: Iberian Peninsula; BRT: British Isles; M+WE: Middle and Western Europe; MED: Circum‐Mediterranean region; EEU: Eastern Europe; CAL: California; NAM: North America except California; M+SA: Middle and South America; AUS: Australasia; AFR: Africa

The average number of different alleles at a locus varied among regions from 1.4 to 3.6 (mean across regions 2.6), and the percentage of polymorphic loci per population ranged from 30 to 100, mean 81.7. Observed heterozygosity *H*_0_ was zero in AFR or near zero in BRT, M + SA, and AUS (0.001). *H*_0_ over all loci was 0.003 (Table 1), and expected heterozygosity *H*_e_ over all loci was 0.23 (Table 1). *F*_IS_, the degree of inbreeding within populations (=loci), was high, 0.980 across populations varying from 0.947 to 0.99 (Table 2) as was the inbreeding coefficient relative to the total *F*_IT_ (ranging from 0.95 to 0.998, mean 0.982, Table 2). The degree of population divergence *F*_ST_ varied from 0.088 to 0.186 and was 0.134 across loci (Table 2). The pairwise calculated fixation indices (*F*_ST_), a measure of population differentiation, revealed population differentiation between regions (Table 3). The *F*_ST_ values were highest between ASIA and CAL (0.137), and AFR and NAM (0.132) and lowest between AUS and IBE (0.014), and CAL and IBE (0.016) (Table 3). Genetic diversity within regions (AMOVA) is presented in Figure 3.

**Table 1:** Measurements of genetic variation within *C. bursa‐pastoris* regions

Region	*n*	*N*_a_	s *N*_a_	*P*	*N*_e_	s *N*_e_	*H*_0_	s *H*_0_	*H*_e_	s *H*_e_
IBE	1,102	3.0	0.30	100	1.428	0.193	0.002	0.001	0.213	0.073
BRT	165	2.2	0.33	70	1.391	0.134	0.001	0.001	0.224	0.068
M + WEU	1,641	3.6	0.37	90	1.572	0.185	0.004	0.002	0.290	0.073
CIR	592	2.9	0.28	90	1.491	0.139	0.004	0.003	0.278	0.064
SCN	468	2.3	0.26	90	1.378	0.192	0.003	0.002	0.202	0.060
EEU	677	3.2	0.44	100	1.550	0.166	0.005	0.003	0.291	0.070
ASIA	281	3.0	0.37	80	1.663	0.238	0.006	0.004	0.301	0.081
CAL	1,203	2.4	0.16	100	1.222	0.070	0.006	0.002	0.158	0.045
NAM	189	2.3	0.34	70	1.574	0.160	0.006	0.003	0.298	0.075
M + SA	423	2.1	0.23	80	1.362	0.131	0.001	0.001	0.212	0.064
AUS	943	2.4	0.31	80	1.434	0.187	0.001	0.000	0.219	0.074
AFR	392	1.4	0.22	30	1.134	0.092	0.000	0.000	0.080	0.053
Total	8,076	2.6	0.1	81.7	1.433	0.047	0.003	0.001	0.232	0.019

Abbreviations: *H*_0_, observed heterozygosity; *H*_e_, expected heterozygosity; *n*, number of individuals per region; *N*_a_, average number of different alleles at a locus; *N*_e_, effective number of alleles; *P*, percentage of polymorphic loci; s *H*_0_, standard error of observed heterozygosity; s *H*_e_, standard error of *H*_e_; s *N*_a_, standard error of *N*_a_; s *N*_e_, standard error of *N*_e_.

**Table 2:** Measurements of genetic variation and *F*‐statistics

Locus	*n*	*N*_e_	*H*_0_	*H*_e_	Fixation index	*F*_IS_	*F*_IT_	*F*_ST_
AAT1	2.209	1.213	0.003	0.131	0.976	0.947	0.95	0.088
AAT2	2.292	1.286	0.001	0.169	0.993	0.99	0.991	0.121
AAT3	3.292	1.667	0.007	0.302	0.978	0.975	0.979	0.186
GDH2	2.417	1.590	0.001	0.336	0.998	0.998	0.998	0.154
LAP3	2.625	1.410	0.004	0.213	0.980	0.989	0.992	0.119
Mean	2.567	1.433	0.003	0.230	0.986	0.980	0.982	0.134

Abbreviations: *F*_IS_, inbreeding coefficient; *F*_IT_, overall inbreeding coefficient; *F*_ST_, degree of population divergence; *H*_0_, observed heterozygosity; *H*_e_, expected heterozygosity; *n*, number of alleles; *N*_e_, effective number of alleles.

**Table 3:** Pairwise population Fst values of C. bursa‐pastoris between regions. AFR: Africa

	AFR	ASIA	AUS	BRT	CAL	MED	EEU	IBE	M+SA	M+WE	NAM	SCN
AFR	0											
ASIA	0.14	0										
AUS	0.063	0.107	0									
BRT	0.119	0.062	0.07	0								
CAL	0.06	0.137	0.037	0.091	0							
MED	0.068	0.058	0.038	0.021	0.063	0						
EEU	0.108	0.036	0.083	0.053	0.101	0.045	0					
IBE	0.044	0.106	0.014	0.082	0.04	0.054	0.09	0				
M+SA	0.067	0.109	0.045	0.065	0.016	0.039	0.076	0.048	0			
M+WE	0.129	0.072	0.108	0.034	0.124	0.033	0.052	0.119	0.082	0		
NAM	0.132	0.104	0.053	0.046	0.066	0.027	0.057	0.064	0.053	0.045	0	
SCN	0.114	0.039	0.097	0.037	0.127	0.051	0.049	0.096	0.099	0.072	0.076	0

Abbreviations: AFR, Africa; AUS, Australasia; BRT, British Isles; CAL, California; EEU, Eastern Europe; IBE, Iberian Peninsula; M + SA, Middle and South America; M + WE, Middle and Western Europe; MED, circum‐Mediterranean; NAM, North America (except California); SCN, Scandinavia. Color shade sequence: red ‐ orange ‐ yellow ‐ green corresponds to decreasing F_ST_ values.

A Mantel test calculated on partitioned data sets for each region, revealed significant, but low correlation between genetic and geographic distances (Table S7). The regression coefficient was highest for SCN (*r* = 0.484, *p* = .001) and EEU (*r* = 0.241, *p* = .001), and lowest in IBE (*r* = 0.042, *p* = .01) and BRT (*r* = 0.069, *p* = .016) .

### 3.4 Global genotype classification

**Figure 4:**
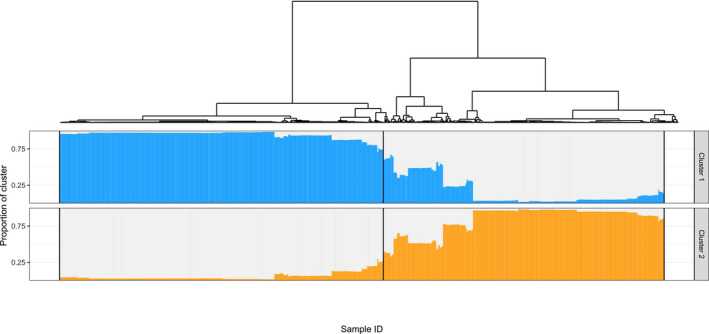
Tree bar plot showing a combination of a hierarchical clustering approach (UPGMA, average linkage) with the results of the Bayesian K‐means algorithm provided with the structure software. Each strip of the bar plot represents a sampled individual and is approximately matched by the respective tips of the hierarchical cluster tree. The central black line across the bar plots indicates the dividing line between the individual samples designated to the respective two main UPGMA clusters

**Figure 5:**
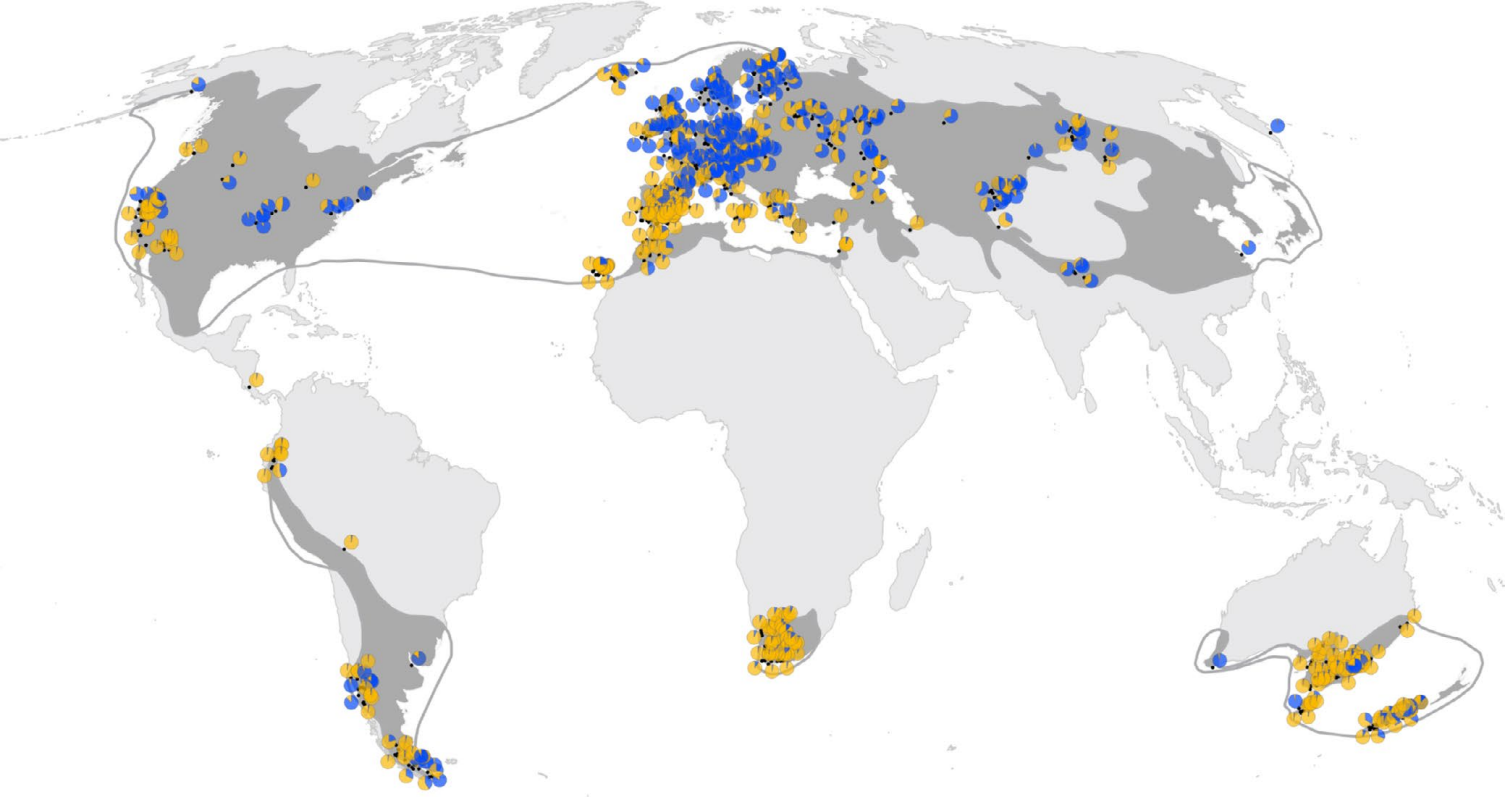
Mapped results of the K = 2 solution of the Bayesian K‐means population structure analysis of *Capsella bursa‐pastoris* sampling sites. Proportional cluster affiliation of the analyzed populations is displayed with pie charts. Blue: estimated proportion of individuals belonging to Cluster 1. Orange: estimated proportion of individuals belonging to Cluster 2.Black dots: sample locations, grey areas: distribution of *C.bursa‐pastoris* based on data compiled by EW (CDH, 2018)

**Figure 6:**
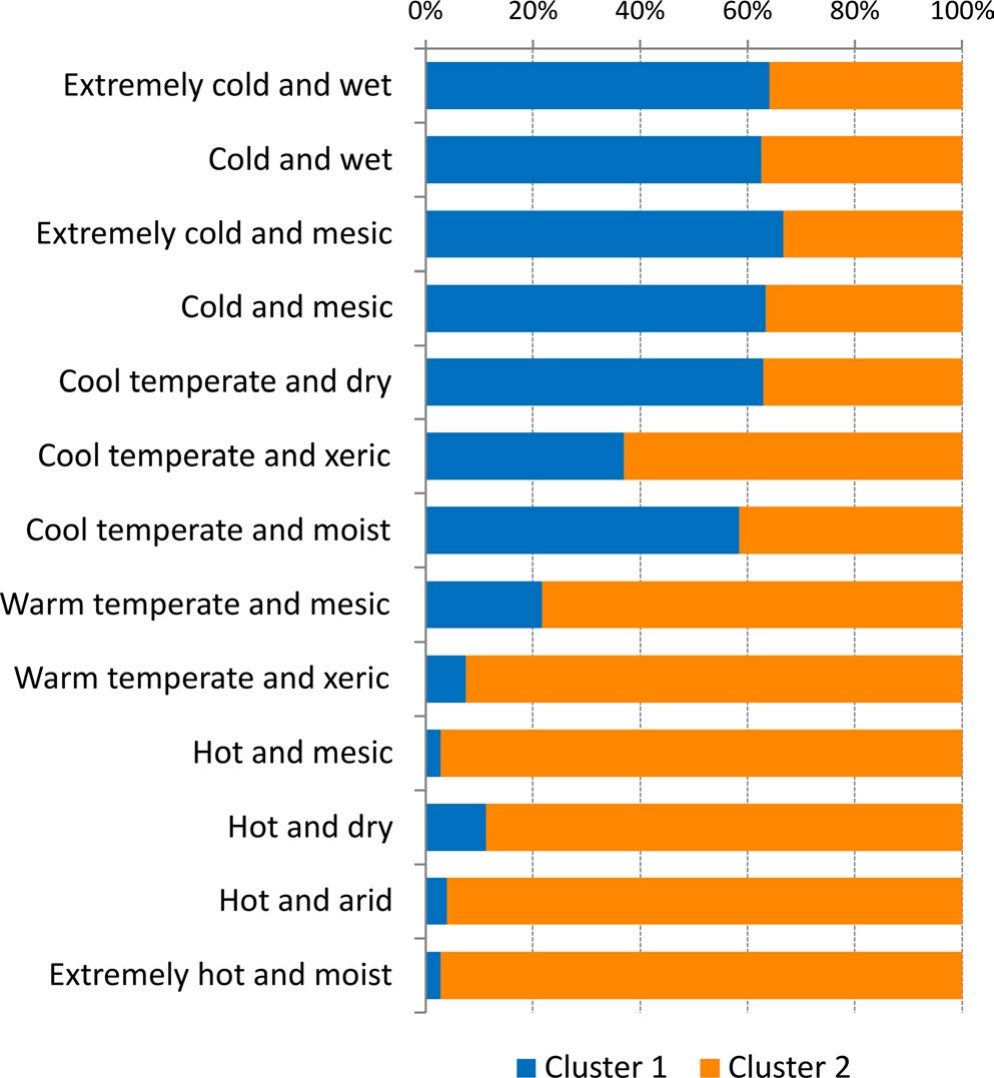
Proportional cluster affiliation of *Capsella bursa‐pastoris* assigned to GEnS (Metzger et al., 2013) climate type regions. Blue: Cluster 1. Orange: Cluster 2

The Bayesian population admixture analysis revealed two main clusters and identified a large proportion of mixed populations (Figure 4). Log probabilities [ln Pr(*X*|*K*)] and plots of ∆*K* obtained by using the software *structure harvester* (Earl & von Holdt, 2012) showed the strongest decrease in inertia for *K* = 2 (∆*K* = 25.19) and are provided in Figure S1. For the alternative solutions of *K* = 3 and *K* = 4 (Figure S2), lower ∆*K* values (2.67 and 1.57, respectively) point to an increase in between‐run differences in population assignments to the respective cluster groups. These between‐run correlation values decrease from 0.988 for *K* = 2 to 0.798 and 0.785 for *k* = 3 and *K* = 4, respectively. Mapping the *K* = 2 solution revealed genetic affiliation of populations of warm–temperate, semiarid versus cool–temperate, semihumid to humid origin to different genetic clusters and the highest admixture of populations in regions with cool temperate, yet xeric climate (Figures 5 and 6). The Mediterranean MMG is the determinant factor for this clustering, 86.2% of the MMG’s *Aat* component, 96.9% of its *Gdh* component, and 92.4% of the *Lap* component are assigned to cluster 2. In the native Eurasian range, Cluster 1 (blue) is predominantly distributed in cool and temperate, more continental climatic regions, and Cluster 2 (orange) in hot to warm and dry mediterranoid climate regions (Figure 6).

The bipartitioning of *C. bursa‐pastoris* is confirmed via independent clustering approaches using different distance measures. Binary data clustering based on combinations of Jaccard, Tanimoto‐Rogers, and Gower similarities and Ward, average (Figure 4), and complete linkage clustering algorithms resulted uniformly in two clusters as the best partitioning solution. The phi coefficients of contingency between the structure bipartitioning and the nine respective alternative solutions ranged between 0.745 and 0.796 with a mean agreement of 89.75%.

For the AMOVA, we used as factors the regions, the cluster affiliation as derived from population admixture analysis, and native versus introduced range part as the grouping variables (Table 4). Genetic variation among groups of populations was highest when partitioning the samples into two groups according to their cluster affiliation (27%) and lowest according to whether samples were from native or introduced regions (11%, Table 4). Using region as a grouping variable, sample size‐corrected SSWP/*n* − 1 values detected ASIA and NAM as being the most diverse regions, followed by EEU, M + WE, and MED (Figure 3). Among the least diverse population regions were CAL and AFR (Figure 3).

**Table 4:** Results of the analyses of molecular variance (AMOVA) of *C. bursa‐pastoris*

Source of variation	*df*	SS	Variance	%
Regional analysis				
Among populations	11	4,297.875	0.301	21%
Within populations	16,140	18,616.527	1.153	79%
Total	16,151	22,914.402	1.454	100%
Cluster affiliation				
Among populations	1	3,496.531	0.438	27%
Within populations	16,150	19,418.699	1.202	73%
Total	16,151	22,915.230	1.640	100%
Native versus introduced				
Among populations	1	1,258.671	0.164	11%
Within populations	16,150	21,655.731	1.341	89%
Total	16,151	22,914.402	1.505	100%

Abbreviations: %, percentage of variance; *df*, degrees of freedom; SS, sums of squares.

All *p*‐values were .01.

### 3.5 Classification tree analysis

The different classification approaches revealed similar results, both in classification agreement with the genetic bipartitioning (*K* = 2) and in the identification of the most discriminating macroclimatic variables. The *rpart* approach obtained 79.6% proportional agreement with the structure bipartitioning, while the ctree approach in *party* and the *randomForest* approach both revealed 82.8% agreement, respectively. With a mean agreement of 81.77% (*SD* 1.84%), the *K* = 2 solution was distinctly better supported eco‐geographically than the *K* = 3 and *K* = 4 solutions (69.48, % *SD* = 0.64% and 64.61%, *SD* = 3.65%, respectively).

The *rpart* package in R identified isothermality (Bio3: CL‐01 < 36.3% < CL‐02) in combination with winter coldness (Bio6: CL‐01 < 1.9°C < CL‐02) and moist quarter temperature (Bio8: CL‐01 < 7.9°C < CL‐02) as most distinguishing variables. Conditional inference classification in *party* revealed again isothermality (Bio3: CL‐01 < 36.3% < CL‐02) as most important, accompanied by humidity (Bio13: mean precipitation of wettest month [CL‐01 < 89 mm < CL‐02]) and summer moisture (Bio18: CL‐01 > 172 mm > CL‐02) as discriminatory predictors. Winter coldness (Bio6: CL‐01 < 1.7° < CL‐02) was identified with nearly the same threshold value in this approach. Similarly, the *randomForest* approach identified Bio3, Bio6, and Bio18 as the most important variables for a successful classification of the samples into the genetically derived cluster partitioning.

### 3.6 Distribution modeling

**Figure 7:**
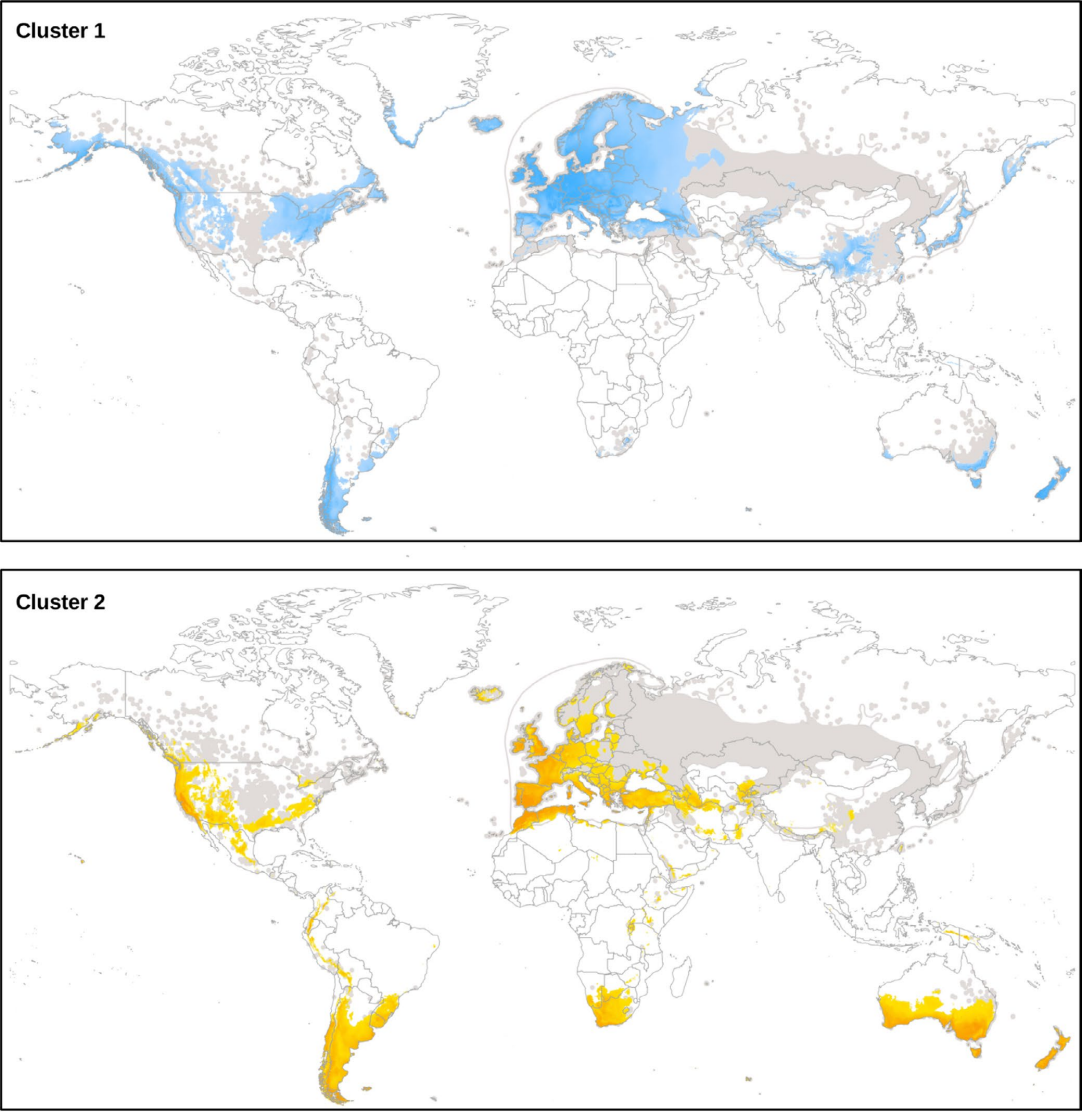
Species distribution models (SDM) for *Capsella bursa‐pastoris*: Occurrence of the obtained clusters projected to the current climate (MaxEnt). Both cluster distributions could be modeled with high accuracy based on macroclimatic data. Grey areas: distribution of *C.bursa‐pastoris* based on data compiled by EW (CDH, 2018)

For the SDM, the feature combination of L + Q + P and a regularization value of 1.1 was obtained as the best configuration option to avoid overfitting. With AUC test values of 0.94 and 0.95, respectively, both cluster distributions could be modeled with high accuracy based on macroclimatic data. The distribution for the two clusters solution resulted in a quite distinct geographical pattern (Figure 7), overlapping only in the oceanic regions of Western Europe, Chile/Argentina, and Tasmania/New Zealand. Cluster 1 (CL‐01) tends to have a more cool–temperate semihumid distribution, while Cluster 2 (CL‐02) is situated in warm–temperate, semiarid–mediterranoid regions (Figures 5 and 6), yet when modeled based on macroclimatic data, this pattern gets even more distinct (Figure 7). The most important variables for the successful prediction of Cluster 1 (CL‐01) were related to summer aridity (Bio14: precipitation of driest month) and winter coldness (Bio11: mean temperature of coldest quarter). The distribution of the “mediterranoid” Cluster 2 (CL‐02) could be best modeled by winter conditions (Bio19: precipitation of the coldest quarter and Bio11: mean temperature of coldest quarter). Here, also temperature seasonality (Bio3: isothermality) was an important predictor.

## 4 DISCUSSION

For about 1,000 years, human activities transported vascular plant species in large numbers and at increasing rates between and within continents (e.g., Crosby, 1986). Introduction dynamics and patterns of plant migration are mostly known in general terms only. *Capsella bursa‐pastoris* might be a “role model” for the history of worldwide weed dispersal by European colonists. We here analyze the global genetic diversity patterns of *C. bursa‐pastoris* and discuss the invasion process in terms of colonization history and adaptation.

### 4.1 Global genetic diversity of *Capsella bursa‐pastoris*

Measurements of genetic variation and *F*‐statistics were performed for all samples without missing data (*n* = 8,076, see chapter 2.3).

The observed heterozygosity *H*_0_ was near zero (Table 1), and the inbreeding coefficients *F*_IS_ and *F*_IT_ indicated a global deficit of heterozygotes within populations. The overall inbreeding coefficient (=fixation index *F*) was near + 1 (0.986) (Table 2). The outcrossing rates, estimated by the *F*‐values (fixation index), *t* = (1 − *F*)/(1 + *F*) (Brown & Weir, 1983), varied between 0% and 1%. Estimation of outcrossing rates, however, depends on the character used and on the estimation procedure. Shull (1929) based on morphological traits inferred outcrossing rates for *C. bursa‐pastoris* of 1%–2% under field conditions. Maximum‐likelihood estimates based on allozyme polymorphism and progeny analyses using the procedure of Brown et al. (1975) revealed outcrossing rates for *C. bursa‐pastoris* between 0% and 10% (Hurka et al., 1989) and may come up to nearly 20% (unpublished results). Thus, data for *C. bursa‐pastoris* indicate a predominantly selfing but flexible mating system. Outcrossing rates may vary considerably.

Polymorphisms within populations expressed by the percentage of polymorphic loci varied between populations from 30 up to 100 (Table 1). Similar values were recorded in previous allozyme analyses of *C. bursa‐pastoris* (Neuffer et al., 2011).

A mean value of *F*_ST_ = 0.134 across all loci indicated significant genetic differentiation between populations, and *F*_ST_ values across single loci varied from 0.09 to 0.19 (Table 2). These results agree with the expectation for a predominantly selfing annual plant. Cornille et al. (2016) reported *F*_ST_ values of the same magnitude for pairwise genetic differentiation between their three clusters.

Bottleneck and founder events, gene flow events, and properties of the soil seed bank in connection with the great colonizing ability of *C. bursa‐pastoris* are another source of interpopulation genetic differentiation (Hurka, 1984, 1990). Below, the genetic differentiation pattern is further analyzed regarding the evolutionary and colonizing history of *C. bursa‐pastoris*.

### 4.2 Biogeography of genetic diversity

#### 4.2.1 Worldwide distribution of alleles

To exclude possible sampling errors, we concentrated on the more common alleles to illustrate allele frequencies (Figure 1, Table S3). They are distributed worldwide indicating that all of the common alleles have been introduced from the native into the non‐native regions (Figure 1). Allozyme diversity in the native range is more pronounced in western Eurasia than in eastern Eurasia (Figure 1). Some of the allozymes are more or less evenly distributed throughout the world (Figure 1, Table S3), whereas frequencies of others varied conspicuously between the geographical regions (Figure 1, Table S3). Source populations are often located in Middle and Western Europe, in the circum‐Mediterranean region, in the Iberian Peninsula, and in the British Isles (Figure 1, Table S3). For details, see Results 3.1.

It appears that allozyme frequencies reflect, to some extent, the history of distribution areas. This is corroborated by the pairwise population *F*_ST_ values between regions (Table 3). None of the values exceeds 0.15 supporting moderate differentiation between native and introduced ranges in general. Rather low *F*_ST_ values between introduced and native regions (ca. ≤0.06) may indicate the main source regions. This is in good agreement with weed introduction history (see “Biological Expansion of Europe,” Crosby, 1986; regarding *Capsella* see Neuffer & Hurka, 1999 for North America; Neuffer et al., 1999 for South America; Harvey & Sonder, 1860 and Marais, 1970 for South Africa; Kloot, 1983 for Australasia).

Pairwise *F*_ST_ values between the geographical regions within the native Eurasian range are mostly low (*F*_ST_ < ca. 0.06) indicating considerable gene pool exchange between the regions. Remarkably, the genetic differentiation between the Iberian Peninsula (IBE) and all other Eurasian regions except the circum‐Mediterranean region (MED) is more pronounced (*F*_ST_ > 0.09, Table 3). This can be explained by the geographical position of the Iberian Peninsula and the Pyrenean mountain chain making gene exchange with northern areas more difficult.

#### 4.2.2 Worldwide distribution of multilocus genotypes

Comparing the frequency of isozyme genotypes instead of alleles alone strongly supports the conclusions drawn above (Table S4). The isozyme genotype distribution patterns argue for intercontinental introduction routes from native Mediterranean and temperate regions into the colonized continents New World, Africa, and Australasia. This is in agreement with the respecting colonizing history (see above).

The geographical distribution of genotype diversity within the native Eurasian range is surprising. Genotype diversity was highest in M + WE and EEU but very low in BRT (only ca. 25% of M + WE) and more or less half of that of the other Eurasian regions (Figure 2, Table S6). This is also mirrored by the AMOVA statistics (Figure 3). Intracontinental migration routes can help to explain this pattern, assuming two centers of initial diversity, namely nemoral Asia and the Mediterranean region. Migrations from east to west and from south to north, probably in postglacial times, overlapped in M + WE and EEU and thus enriched genotype diversity in these regions. Population structure analyses, which showed admixture of two clusters in continental Europe, support this scenario (Figure 5). The British Isles, because of their geographically isolated position, received only part of the diversity.

#### 4.2.3 Isolation by distance

Isolation by distance is another source of genetic variation between populations (Mantel's test). A statistically significant linear correlation between genetic and geographic distances exists for all regions (Table S7). The correlation was positive except for Middle and South America (M + SA). However, the slopes of the regression lines and the coefficient of determination *R* ^2^ are very low. Less than 6% (0.06 > *R* ^2^ > 0.00) of the genetic distances among populations within the geographical regions are explained by the geographical distance. Exception is Scandinavia with 23% (Table S7).

### 4.3 Two lineages within *Capsella bursa‐pastoris*

It appears that, based on multilocus isozyme genotypes, *C. bursa‐pastoris* is split into two lineages, or clusters, one occurring in Mediterranean climate regions and the other occurring in temperate climate regions. The two lineages are robust across methods ranging from Bayesian clustering to simple distance measures and clustering approaches for binary data. This bipartition is also corroborated by independent macroclimatic data (Figures 6 and 7) and might point to a lasting parental legacy in terms of the climatic niches of the ancestral diploid lineages, from which the contemporary species *C. grandiflora* and *C. orientalis* originated. However, the two lineages might also be the result of an early diversification after the origin of *C. bursa‐pastoris*.

Shull (1929) described a new species, *Bursa* (=*Capsella*) *occidentalis*, which, however, was never recognized let alone accepted. Plants belonging to this taxon are early flowering and display some leaf characteristics varying from other *Capsella* provenances. Shull recorded it from Arizona, California, Hawaii, Peru, Chile, Argentina and Uruguay, and noted, “how closely the range of this species […] agrees with the region occupied by the Spanish settlers in America. It seems probable that there is a causal relation between these two distributions.” Apparently, Shull's *C. occidentalis* belongs to the Mediterranean lineage shown here. Crossing experiments between populations with typical Mediterranean and temperate genotypes indicated that the success rate of crossing is restricted and even failed in the case where the mother plant was of the Eurosiberian type (Linde, 1999, unpublished), leading to the assumption that there is some incompatibility between the two lineages.

Based on a genome‐wide set of SNP markers, Cornille et al. (2016) detected three clusters in *C. bursa‐pastoris*: a Europe/Russia cluster (termed “EUR”) extending from Western Europe to the Russian Far East, a cluster with main distribution in eastern and southern Mediterranean regions (termed “Middle East,” ME), and a cluster in southeastern China (termed “ASI”). The term “Middle East” for the mainly Mediterranean distributed cluster is misleading. By far, the largest part of what is traditionally defined as Middle East is not sampled. The six “true” Middle East sample sites of Cornille et al. (2016) are located in typical Mediterranean climate regions near the eastern shores of the Mediterranean Sea (1 in Turkey, 3 in Syria, 1 in Israel, and 1 in Jordan). Cornille et al. (2016) point out that their study missed a large sampling area of *C. bursa‐pastoris* populations in Middle Asia, which is likely a key region to disentangle the origin of *C. bursa‐pastoris*. Between the most eastern locality of the ME cluster (in Syria) and the most western locality in the ASI cluster (in Qinghai, China) is a sampling gap of ca. 5.400 km, and within the EUR cluster is a sampling gap of ca. 4.300 km between the most eastern European site (Voronezh) and the most western Asian site (near Irkutsk). This sampling gap calls for caution in inferring demographic histories of the three clusters as discussed by Cornille et al. (2016).

It appears that our Cluster 1 corresponds to the European/Russian cluster of Cornille et al. (2016), and Cluster 2, to the “Middle East” cluster. Reason why we did not detect the third ASI cluster might be missing samples in southeastern China in our study. However, the ASI cluster is also distributed in the Mediterranean region, where we sampled well enough but did not find it, nor did we detect a third cluster in the introduced ranges in contrast to Cornille et al. (2016) who report the ASI cluster also from the USA. This lends support to the assumption that the ASI cluster is below the isozyme detection threshold. While the sensitivity of the isozyme approach is clearly limited, an important advantage of our study is the much better sampling coverage of the source and target areas of the transatlantic colonization of the new world.

### 4.4 Analyses of variance, AMOVA

We used the AMOVA procedure to calculate the level of differentiation among different populations. It allows hierarchical partitioning of genetic variation between populations and regions. We calculated genetic differences between all 12 geographical regions, differences between native and non‐native regions, and differences between the two lineages within *C. bursa‐pastoris*.

Only 11% of the genetic variation can be attributed to the differences between native and non‐native regions implying that the difference between native and introduced areas is small (Table 4, Figure 3). The genetic differences between the 12 geographical regions exceeded that among native versus non‐native regions. It amounted to 21% (Table 4, Figure 3). Highest genetic differentiation with 27% was between populations affiliated to the two lineages/clusters (Table 4). Thus, the AMOVA corroborates the worldwide geographical structuring of the genetic variation of *C. bursa‐pastoris*, supports the two lineages within the species, and confirms the small genetic differentiation between native and non‐native regions.

### 4.5 Colonization and adaptation

We recorded all common multilocus genotypes detected in Europe also in the introduced continents (Table S5) and concluded that the variable European *Capsella* gene pool was nearly completely introduced into the other continents (see also Table 3). This provides evidence for multiple introductions instead of rearrangements of a single or few introduced genotypes in the newly colonized regions. However, since the temperate lineage and the Mediterranean lineage of *C. bursa‐pastoris* are obviously linked to climate parameters (Figure 6), it is reasonable to assume that preferences for different climatic macrodata (Figure 7) may act as an environmental filter on colonization success. California is a good example. Mediterranean climate regions in California are nearly exclusively colonized by the Mediterranean *Capsella* lineage whereas the temperate lineage colonized the snowy‐forest and coastal redwood climate regions (Neuffer & Hurka, 1999; see also Figures 5 and 6). This distribution pattern is not in accordance with the spread of European colonizers throughout California but argues for environmental constraints of the establishment success of the introduced genotypes. It has been shown in previous *Capsella* studies that variation in isozymes is correlated with ecologically important life‐history traits, such as flowering time and growth form parameters (Neuffer & Hoffrogge, 1999; Neuffer & Hurka, 1999). This correlation can be explained, at least partly, by linkage of isozyme loci to life‐history traits. Linde et al. (2001) found three major QTL controlling flowering time differences among ecotypes, which are linked to isozyme loci. These linkage groups correspond to single chromosomes.

Multiple introductions are very common features of successful invasions (Bossdorf et al., 2005; Dlugosch & Parker, 2008). While some successful colonizers arrive well suited to new environments, the success of others appears to depend on rapid local adaption (Bock et al., 2015). Populations adapt to novel environments in two ways: selection on pre‐existing standing variation and selection on new, de novo mutations. One source of standing variation in the introduced range is admixture, the mixing of historically isolated gene pools (Dlugosch et al., 2015). It is the result of multiple introductions and introgression among diverse genotypes from geographically structured populations in the native range thereby generating heterozygosity (Keller et al., 2014). In our *Capsella* study, admixture between the temperate and Mediterranean lineages has been demonstrated (Figure 4), but whether this is a significant source of new standing variation in the introduced range is questionable. Nevertheless, in nearly all regions, we recorded “endemic” genotypes (Table S6, Figure 2). They may be the outcome of admixture or may be de novo mutations, but this cannot yet be determined. Since the degree of endemism calculated as the ratio of endemic to overall genotypes is unrelated to sampling intensity (Pearson's *r* = 0.52, *p* = .085), sampling bias seems unlikely. The generally relatively low number of (frequent) genotype endemism in regions of post‐Columbian colonization points to a limited importance of new genotypes. It seems that natural selection in invaders relies mainly on standing variation (Bock et al., 2015), and it seems that *C. bursa‐pastoris* is no exception. The colonizing success of *C. bursa‐pastoris* in the introduced ranges seems to be based on the presence of introduced preadapted genotypes/pre‐existing standing variation rather than on selection for adaptive genetic variation after the introduction.

## 5 CONCLUSION

The global biogeography of genetic variation of *C. bursa‐pastoris* at the isozyme level is clearly geographically structured and is split into two lineages: one distributed predominantly in Mediterranean climate regions and the other predominantly in temperate climate regions. The distribution pattern of these lineages in native Eurasia can be explained by the evolutionary history of *C. bursa‐pastoris* and intracontinental migration in prehistoric times, whereas intercontinental migration in historic times (predominantly post‐Columbian) explains the geographical patterns in the introduced ranges, which mirror the history of weed introduction into the continents. Multiple independent introductions of genotypes from different sources and climate regions are obvious. Since we detected nonrandom, predictable niche properties of the two lineages, we suggest involvement of environmental filtering and hypothesize that most of the successfully colonizing *Capsella* genotypes were preadapted and found their respective matching niches in the colonized ranges. These assumptions need to be tested experimentally, since our macroscale approach is not sufficient to draw clear causal or mechanistic conclusions.

## CONFLICT OF INTEREST

The authors have no conflicts of interest to declare.

## AUTHOR CONTRIBUTIONS

**Christina Wesse:** Software (equal); Validation (equal); Visualization (equal); Writing‐original draft (equal); Writing‐review & editing (equal). **Erik Welk:** Software (equal); Validation (equal); Visualization (equal); Writing‐original draft (equal); Writing‐review & editing (equal). **Herbert Hurka:** Conceptualization (equal); Data curation (equal); Investigation (equal); Methodology (lead); Resources (equal); Supervision (equal); Writing‐original draft (equal); Writing‐review & editing (equal). **Barbara Neuffer:** Conceptualization (equal); Data curation (equal); Funding acquisition (lead); Investigation (equal); Methodology (equal); Project administration (lead); Resources (equal); Supervision (lead); Writing‐original draft (equal); Writing‐review & editing (equal).

## Supporting information

Supplementary Material

## Data Availability

The isozymes data sets generated during and/or analyzed during the current study are available from the corresponding author on reasonable request. Much of the data generated or analyzed during this study are included in this published article and its supplementary information files. The data that support the findings of this study are deposited in Dryad (https://doi.org/10.5061/dryad.rxwdbrv5c).
