# Supplementary material for: Geographical pattern of genetic diversity in Capsella bursa‐pastoris (Brassicaceae)—A global perspective
Source: Ecol Evol. 2020 Nov 13;11(1):199–213. doi: 10.1002/ece3.7010 (PMC7790636; doi:10.1002/ece3.7010)
Supplement: Supplementary file 1 — Supplementary Material [file ECE3-11-199-s001.docx]

**Supplementary Files**

**Supplementary Table S1** Supplementary Table S1a Provenances of Capsella bursa-pastoris populations. Country of origin is indicated by the international KFZ acronym. a) all provenances. b) provenances of only complete multilocus dataset

**Supplementary Table S2** Allele frequencies within different species of the genus Capsella at the loci Aat1, Aat2, Aat3, Gdh2, Lap3. Alleles are characterised by numbers after the corresponding locus. AAT: Aspartate aminotransferase, GDH: Glutamate dehydrogenase; LAP: Leucine aminopeptidase. For details see text.

**Supplementary Table S3** Frequencies of *Capsella bursa-pastoris* alleles between and within regions. For characterisation and nomenclature of alleles see Supplementary Table S1. IBE: Iberian Peninsula. BRT: British Isles. M+WE: Middle and Western Europe. MED: Circum-mediterranean. SCN: Scandinavia. EEU: Eastern Europe. CAL: California. NAM: North America (except California). M+SA: Middle and South America. AUS: Australasia. AFR: Africa.

**Supplementary Table S4** Number and frequencies of all recorded genotypes of *Capsella bursa-pastoris* at single loci and at multicocus associations. Green: Genotypes with frequencies > 10% per region for genotypes with total frequencies > ca. 10%. For nomenclature of loci see Supplementary Table S1, and for regions Supplementary Table S2.

**Supplementary Table S5a** Frequencies of complete multilocus genotypes of Capsella bursa-pastoris corresponding between regions. The 18 most common genotypes of 383 are shown in detail (the rest summarized in „others“). Frequencies depicted with .000 are <.001. Entries with dash whenever allele not detected. n number of individuals studied. †: Multilocus Mediterranean Genotype (MMG). IBE: Iberian Peninsula. BRT: British Isles. M+WE: Middle and Western Europe. MED: Circum-mediterranean. SCN: Scandinavia. EEU: Eastern Europe. CAL: California. NAM: North America (except California). M+SA: Middle and South America. AUS: Australasia. AFR: Africa.

**Supplementary Table S5b** Frequencies of complete multilocus genotypes of Capsella bursa-pastoris corresponding within regions. The 18 most common genotypes of 383 are shown in detail (the rest summarized in „others“). Frequencies depicted with .000 are <.001. Entries with dash whenever allele not detected. n number of individuals studied. †: Multilocus Mediterranean Genotype (MMG). IBE: Iberian Peninsula. BRT: British Isles. M+WE: Middle and Western Europe. MED: Circum-mediterranean. SCN: Scandinavia. EEU: Eastern Europe. CAL: California. NAM: North America (except California). M+SA: Middle and South America. AUS: Australasia. AFR: Africa.

**Supplementary Table S6** Genotype diversity of *Capsella bursa-pastoris*. Numbers of different genotypes at single loci and multilocus associations are given in total and per region, in brackets number of ‘endemic’ genotypes. Colors refer to the number of genotypes, the darker the color the higher the number. Order statistics from high (order 1) to low (order 12) is given in the bottom line. For nomenclature of loci see Supplementary Table S1, and for regions Supplementary Table S2.

**Supplementary Table S7** Results of the Mantel test of *Capsella bursa-pastoris* populations. All calculations with 999 permutations.


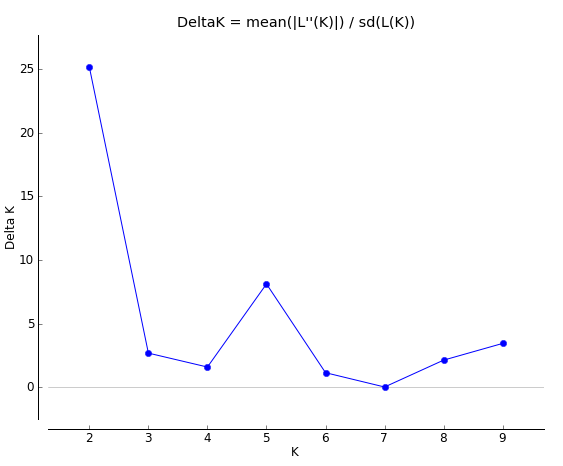


**Supplementary Figure S1** Evanno-plot showing the second order rate of change of the likelihood (∆K) for the respective cluster number as a measure of between-run stability. The largest decrease of ∆K from the K = 2 to the K = 3 solution is based on an increase at K = 3 of between-run variability and accordingly, a higher standard deviation of population designation to a certain group.


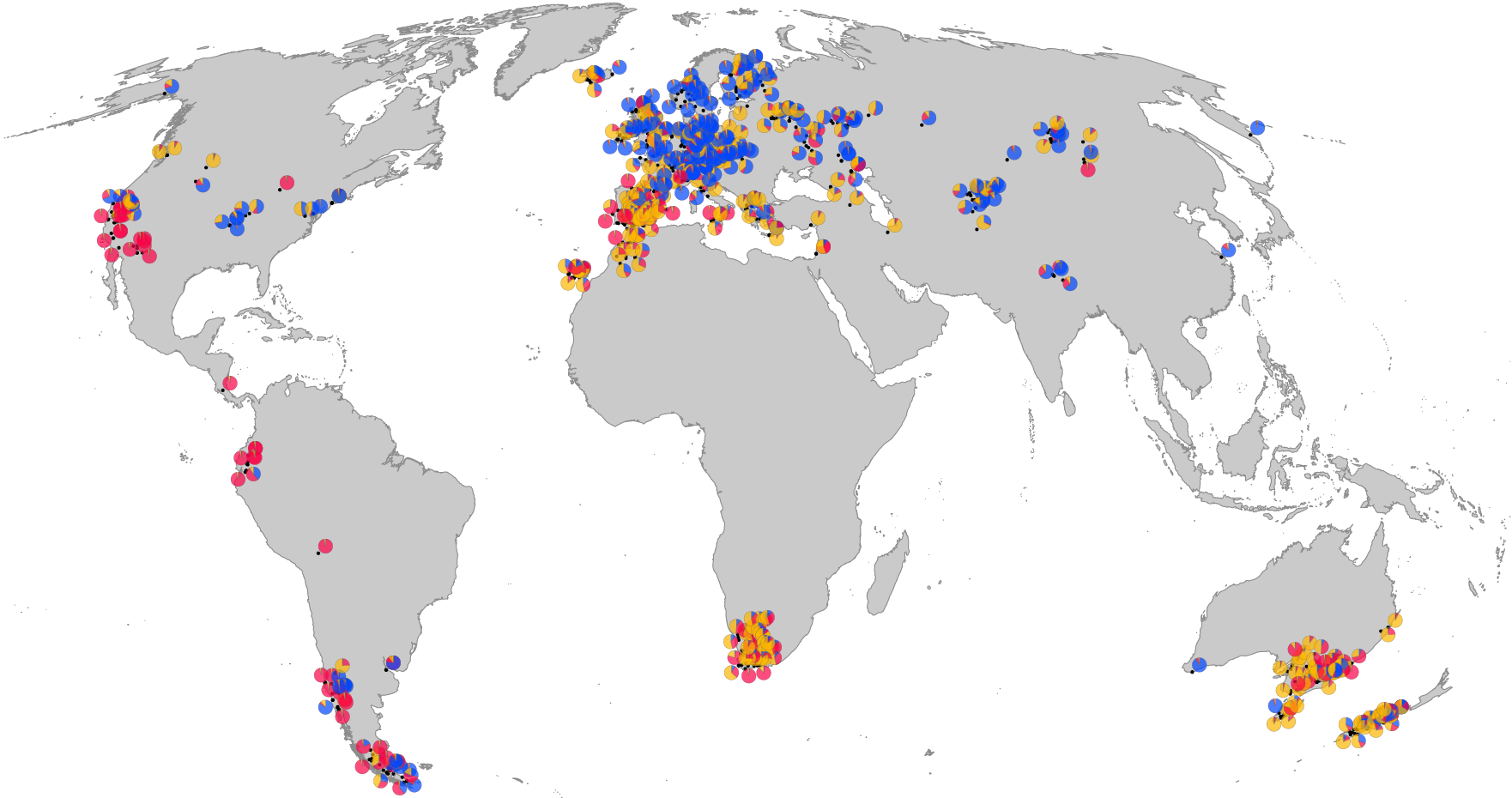


**Supplementary FIGURE 2a** Mapped results of the K = 3 solution of the Bayesian K-means population structure analysis of 8076 individuals across 787 *Capsella bursa-pastoris* sampling sites. Proportional cluster affiliation of the analysed populations is displayed with pie-charts. Blue: estimated proportion of individuals belonging to Cluster 1; Orange: estimated proportion of individuals belonging to Cluster 2. Red: estimated proportion of individuals belonging to Cluster 3. Black dots indicate the exact geographic positions of the sampling localities.


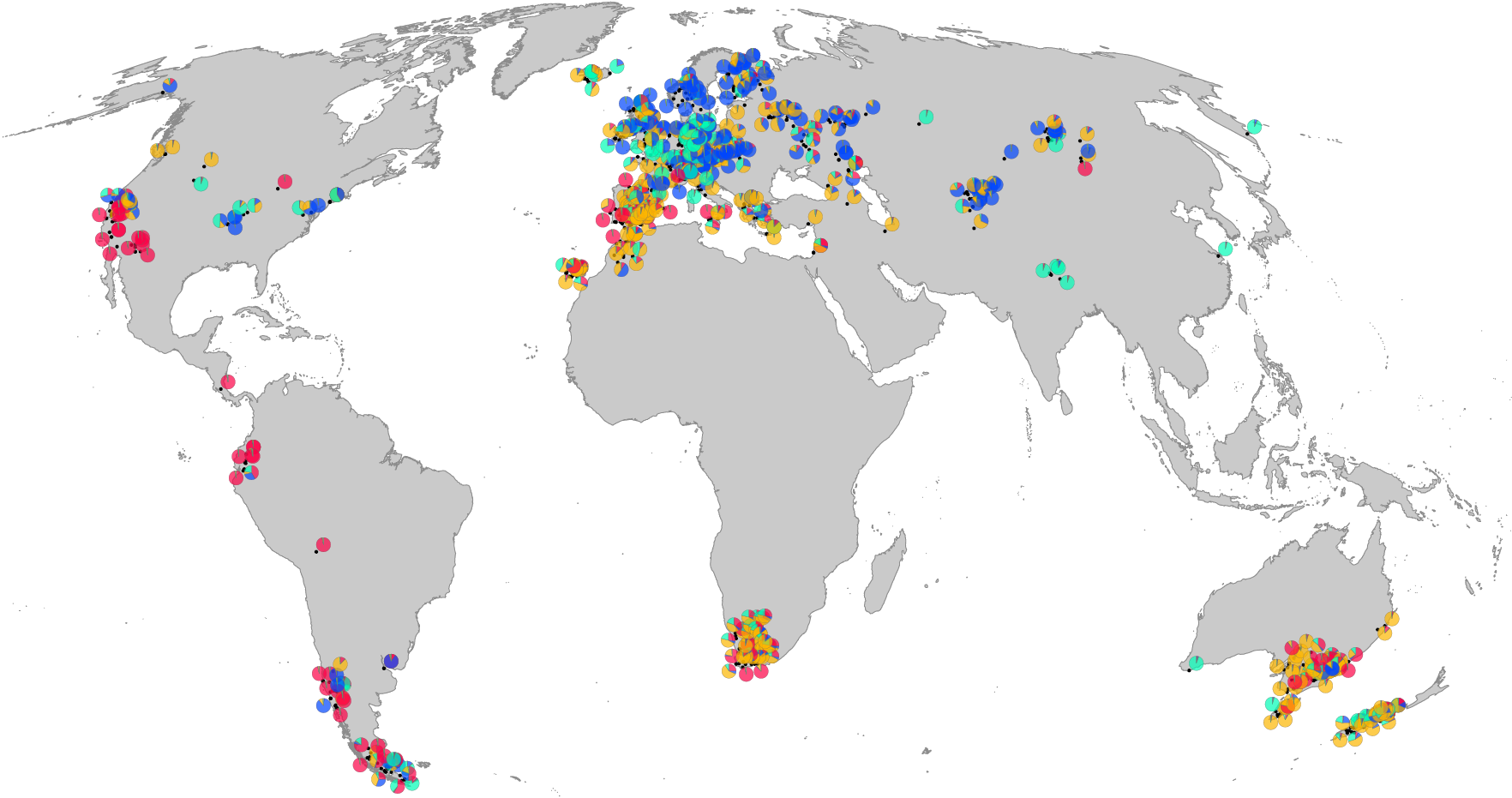


**Supplementary FIGURE 2b** Mapped results of the K = 4 solution of the Bayesian K-means population structure analysis of 8076 individuals across 787 *Capsella bursa-pastoris* sampling sites. Proportional cluster affiliation of the analysed populations is displayed with pie-charts. Blue: estimated proportion of individuals belonging to Cluster 1; Orange: estimated proportion of individuals belonging to Cluster 2. Red: estimated proportion of individuals belonging to Cluster 3. Green: estimated proportion of individuals belonging to Cluster 4. Black dots indicate the exact geographic positions of the sampling localities.
